# Impaired Recruitment of Grk6 and β-Arrestin2 Causes Delayed Internalization and Desensitization of a WHIM Syndrome-Associated CXCR4 Mutant Receptor

**DOI:** 10.1371/journal.pone.0008102

**Published:** 2009-12-01

**Authors:** Peter J. McCormick, Marta Segarra, Paola Gasperini, A. Virginia Gulino, Giovanna Tosato

**Affiliations:** The Laboratory of Cellular Oncology, Center for Cancer Research, National Cancer Institute, National Institutes of Health, Bethesda, Maryland, United States of America; University of Oldenburg, Germany

## Abstract

WHIM (**w**arts, **h**ypogammaglobulinemia, **i**nfections, and **m**yelokatexis) syndrome is a rare immunodeficiency syndrome linked to heterozygous mutations of the chemokine receptor CXCR4 resulting in truncations of its cytoplasmic tail. Leukocytes from patients with WHIM syndrome display impaired CXCR4 internalization and enhanced chemotaxis in response to its unique ligand SDF-1/CXCL12, which likely contribute to the clinical manifestations. Here, we investigated the biochemical mechanisms underlying CXCR4 deficiency in WHIM syndrome. We report that after ligand activation, WHIM-associated mutant CXCR4 receptors lacking the carboxy-terminal 19 residues internalize and activate Erk 1/2 slower than wild-type (WT) receptors, while utilizing the same trafficking endocytic pathway. Recruitment of β-Arrestin 2, but not β-Arrestin 1, to the active WHIM-mutant receptor is delayed compared to the WT CXCR4 receptor. In addition, while both kinases Grk3 and Grk6 bind to WT CXCR4 and are critical to its trafficking to the lysosomes, Grk6 fails to associate with the WHIM-mutant receptor whereas Grk3 associates normally. Since β-Arrestins and Grks play critical roles in phosphorylation and internalization of agonist-activated G protein-coupled receptors, these results provide a molecular basis for CXCR4 dysfunction in WHIM syndrome.

## Introduction

The G protein-coupled receptor CXCR4 and its chemokine ligand stromal cell-derived factor-1 (SDF-1, also termed CXCL12) play essential roles in the development and function of the hematopoietic system [1]–[4]. SDF-1/CXCL12 is constitutively expressed in various adult tissues, including bone marrow, lung, liver, lymph nodes, and skin [5], [6]. In the bone marrow, stromal cells, osteoblasts and endothelial cells are the source of SDF-1/CXCL12 [7]-[9], and contribute to distinct hematopoietic stem cell niches in part through the production of SDF-1/CXCL12 [10]–[14]. Bone marrow myeloid and lymphoid cells express a functional CXCR4.

Homeostatic levels of peripheral blood neutrophils increase during bacterial infections and other forms of stress, and this rise is principally regulated through their dynamic release from the bone marrow to the circulation [15]. Although the biochemical mechanisms underlying this process are incompletely defined, there is compelling evidence that SDF-1/CXCL12 stimulation of CXCR4 is a principal regulator for retention and stress-induced mobilization of myeloid lineage cells from the bone marrow to the blood [16]–[18].

WHIM syndrome is a rare immunodeficiency disorder characterized by papillomavirus-induced **w**arts, **h**ypogammaglobulinemia, recurrent bacterial **i**nfection, and **m**yelokathexis, a type of neutropenia associated with the retention and death of mature neutrophils in the bone marrow [19]–[21]. The majority of patients with WHIM syndrome have been linked to heterozygous genetic mutations in the gene encoding CXCR4 resulting in truncations of the cytosolic carboxy-terminal portion of the receptor and thus co-express the normal and mutant CXCR4 proteins [22], [23]. The most extensive WHIM-associated truncation removes nineteen amino acids from the carboxy-terminus of CXCR4 whereas the least extensive truncation removes only ten amino acids from the carboxy-terminus [21], [22]. Functionally, WHIM-associated CXCR4 mutants display enhanced and prolonged responses to SDF-1/CXCL12, and this CXCR4 gain of function is believed to contribute to increased neutrophil retention to the bone marrow, their reduced release to the peripheral circulation leading to senescence and apoptotic death within the bone marrow [20], [22], [23]. G-CSF, which downregulates expression of the CXCR4 receptor and its ligand SDF-1/CXCL12 [24]–[26], is commonly used to reduce neutropenia in WHIM patients.

A number of studies have investigated the physiologic mechanisms of CXCL12/CXCR4 signaling [23], [27], [28]. In brief, upon ligand binding, CXCR4 becomes phosphorylated on several serine and threonine residues in the cytoplasmic carboxy-terminal tail, recruits a β-Arrestin, which leads to clathrin dependent CXCR4 internalization, ubiquitination, and eventual lysosomal degradation. In spite of general agreement on the sequence of events accompanying CXCR4 signaling and degradation, many questions persist on the biochemical features of many of the steps. In the case of WHIM-associated CXCR4 mutants, it is unclear which step or steps are abnormal. Biochemical studies with WHIM-CXCR4 mutants detected impaired ligand-mediated internalization and calcium ion mobilization in some studies [22], [29], but not others [30]. Signaling dysfunction reflected by altered Erk 1/2 phosphorylation was noted in ligand-activated WHIM leukocytes expressing wild-type (WT) and mutant CXCR4, and was attributed to a transdominant-negative effect of the mutant CXCR4 over the WT CXCR4 [28], [29]. A contribution of β-Arrestin 2 to defective signaling by mutant CXCR4 was suggested by some studies [31]. In addition, altered cell response to SDF1/CXCL12 in mutant mice lacking the G protein-coupled receptor kinase, GRK6, and the discovery of WHIM patients having GRK3 defects and no CXCR4 mutation suggested a contribution of GRKs to signaling defects of CXCR4 mutant receptors [32]–[34].

In the current study, we demonstrate that the WHIM-associated mutant CXCR4 is defective at recruiting β-Arrestin 2 and GRK6 proteins after exposure to the ligand, and displays a delay in ligand-induced internalization, signaling and trafficking in comparison to WT CXCR4.

## Results

### Receptor Internalization of WT and Mutant CXCR4

**Figure 1:**
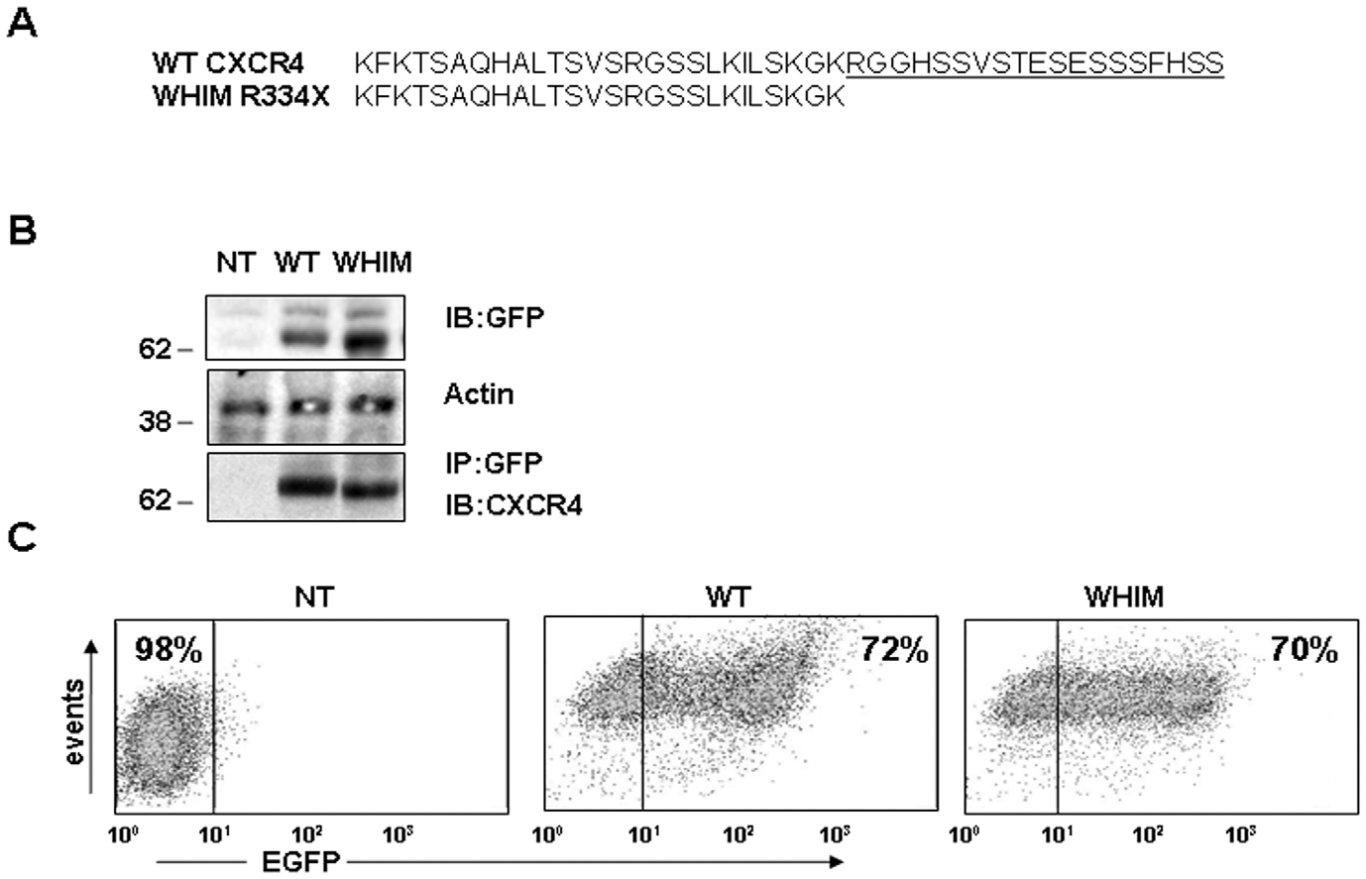
Transduction and stable expression of WT and mutant forms of CXCR4 in HeLa cells. **A**, amino acid sequence of the C-terminal tails of WT CXCR4 and the mutant form of CXCR4 (WHIM R334X) utilized in the current study. **B**, HeLa cells stably transduced with GFP-WT CXCR4 or GFP-WHIM were lysed and analyzed by SDS-PAGE and Western blotting using anti-GFP antibodies or immunoprecipitated with anti-GFP antibody and immunoblotted with anti-CXCR4 antibody. Accuracy of loading was assessed by reprobing the membrane with anti-actin antibodies. **C**, flow cytometric analysis of GFP expression in control (non-transduced, NT) Hela cells and HeLa cells stably transduced with GFP-CXCR4 (WT and WHIM).

To define the molecular basis for WHIM syndrome, we generated HeLa cell lines that stably expressed either wild-type (WT) CXCR4 or a mutant CXCR4 with a 19 amino acid truncation at the carboxy-terminus. This is the most extensive CXCR4 truncation associated with WHIM syndrome [20]–[22], [30], which derived from a frame shift mutation (previously designated WHIM R334X, Figure 1A). Earlier studies have shown that Green Fluorescent Protein (GFP) fusions to the N-terminus of CXCR4 do not disrupt CXCL12/SDF1 ligand binding or receptor function [35]. Therefore, we produced WT and mutant CXCR4 proteins tagged with an N-terminal fusion protein of GFP, which allows visualization of the transfected proteins and distinguishes them from the endogenous protein that is expressed in HeLa cells.

Using antibodies against GFP, Western blot analysis revealed that HeLa cells expressed similar levels of GFP-WT or GFP-WHIM mutant CXCR4 proteins, which displayed their predicted relative molecular weights from the fusion with GFP (Figure 1B). The endogenous CXCR4 (relative size 44–47kDa) was visualized only after prolonged exposure, and was estimated to represent ∼15–20% of the total CXCR4 receptor in the trasnfected cells (data not shown). To establish the identity of the bands recognized by direct Western blotting with anti-GFP antibodies, we used antibodies against GFP to immunoprecipitate the tagged proteins from total cell lysates followed by immunoblotting with antibodies against CXCR4 (Figure 1B). Using FACS analysis, we determined that the percentage of cells expressing GFP-CXCR4 was 67–90% for both constructs and was maintained at this level under selective pressure (Figure 1C, representative results).

**Figure 2:**
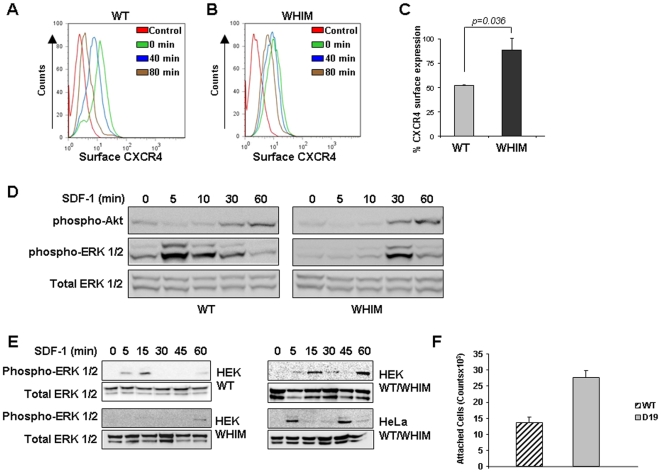
Ligand-induced internalization of WT and WHIM CXCR4 examined by flow cytometry and confocal microscopy. **A–C**, HeLa cells stably expressing GFP-WT CXCR4 or GFP-WHIM CXCR4 constructs were exposed to 25 ng/ml SDF-1/CXCL12 for 0, 40 or 80 min at 37°C and then stained for cell surface CXCR4 using antibodies to GFP to detect the transduced CXCR4 and distinguish it from the endogenous protein; levels of cell surface GFP-CXCR4 were measured by flow cytometry. The red line depicts results from control staining with isotype control immunoglobulin and secondary antibody. Results in A and B reflect a representative experiment showing CXCR4 mean fluorescence intensities at the indicated time-points; results in C reflect the mean (±SD) levels of cell surface CXCR4 (measured by mean fluorescence intensity, MFI) at the 80 min time-point from 3 independent experiments; the results are expressed as a percentage of receptor levels (as measured by MFI) found on the surface at time 0 (0 min). **D**, Hela cells stably expressing GFP-WT CXCR4 or GFP-WHIM CXCR4 were exposed to SDF-1/CXCL12 (25 ng/ml) for the indicated time intervals; cell lysates were separated by SDS-PAGE and analyzed by Western blotting using specific antibodies to phosphorylated Akt and Erk 1/2; for loading controls, membranes were re-probed with antibodies to total Erk 1/2. **E**, HEK 293 cells were transiently transfected with WT, WHIM, or both receptors; HeLa cells were transiently transfected with both WT and WHIM receptors. Transfected cells were exposed to SDF-1/CXCL12 (25 ng/ml) for the indicated times and cell lysates analyzed by SDS-PAGE and Western blotting. **F**, Huvec were plated in monolayers onto 96-well plates, and were activated overnight with 2 ng/ml of TNFα. CFSE-labeled KG1a cells expressing, WT or WHIM CXCR4 were incubated at 5×10^4^ cells per well onto TNFα−preactivated Huvec monolayers in triplicate for 30 min. After removal of non-adherent cells, adherent cells were counted by reading fluorescence at ∼520 nm. The results reflect the mean +/− SD of triplicate wells in a representative experiment performed three times.

Since previous reports have described reduced ligand-induced CXCR4 internalization in cells from patients with WHIM syndrome [28], we tested whether HeLa cells expressing the WHIM-associated CXCR4 mutant show a similar impairment. Using flow cytometry to measure the rate of CXCL12/SDF1-induced CXCR4 internalization, we found that WHIM mutant CXCR4 receptors show a slower rate of ligand-induced internalization compared to WT receptor (Figure 2A and B). At the 80 minutes time-point, the WHIM mutant CXCR4 receptor showed a reduction in the mean internalization compared to the WT receptor (Figure 2C), which was significant (P = 0.036). These quantitative differences in receptor internalization were observed over a wide range of ligand concentrations (10–100 ng/ml, not shown), consistent with cells expressing similar levels of WT or mutant receptors (Figure 1C and Figure 2A, B green line) and with the use of saturating amounts of ligand.

### Receptor Internalization Defects Lead to Delayed MAP Kinase Signaling

CXCR4 can signal through the MAP kinase and Akt pathways [36], [37]. We tested whether ligand-induced signaling from the WHIM mutant CXCR4 receptor is altered compared to that from the WT receptor. Consistent with the results in figures 1 and 2 showing delayed WHIM receptor internalization compared to WT, we found that ligand- induced Erk 1/2, but not Akt, phosphorylation was delayed in HeLa cells expressing the WHIM mutant CXCR4 compared to HeLa cells expressing the WT receptor (Figure 2D). This result is consistent with Lagane et al. who, also found WHIM receptor signaling at later time points [31]. While Erk 1/2 activation by the WT receptor was maximal at 5–15 min, Erk 1/2 activation by the WHIM mutant was maximal at 30–60 min. As a consequence of this difference in kinetics, signaling by a cell consisting of both a WT and a mutant receptor at a 1∶1 ratio would be expected to have a signaling time that would be extended. Such a prediction is consistent with previous reports showing extended Erk 1/2 signaling from WHIM patient cells [31].

To test directly this prediction, we transiently transfected WT and WHIM CXCR4 individually or together in HEK 293 cells and examined the kinetics of Erk 1/2 signaling in response to SDF-1/CXCL12. We selected HEK 293 cells for these experiments because they do not express endogenous CXCR4. As shown in Figure 2E, Erk 1/2 signaling was maximal at 15 minutes in cells expressing the WT receptor, whereas it was only detected at 60 minutes in cells expressing the WHIM receptor. When both WT and WHIM receptors were co-expressed in HEK 293 cells, signaling had a bimodal pattern with a peak at 15 minutes and a second peak at 60 minutes, likely reflective of signaling by the individual receptors. These results support our prediction, and are consistent with the extended signaling observed in patients with WHIM. To confirm that such a bimodal pattern was not unique to HEK 293 cells, we transiently transfected equal amounts of both receptors into HeLa cells. Although the kinetics of signaling were slightly different from those with HEK 293 cells, the bimodal pattern of Erk1/2 signaling was also observed (Figure 2E), providing additional evidence for the presence of prolonged signaling when WT and WHIM receptors are expressed.

We also compared WT and WHIM CXCR4 in attachment assays. We used monolayers of human umbilical vein endotehlial cells (Huvec) as a source of SDF1 bound to cell surface proteoglycans [38]. Since Hela cells attach poorly to Huvec, we transduced the myeloid KG1a (that do not express detectable levels of endogenous CXCR4) with WT and WHIM CXCR4. In experiments not shown, we established that KG1a expressed similar levels of WT and WHIM CXCR4, and that WHIM CXCR4 expressing KG1a displayed a delay in SDF1 induced CXCR4 internalization. As shown in Figure 2E, myeloid cells expressing WHIM CXCR4 displayed a significantly (P<0.05) enhanced attachment to activated Huvec compared to WT control.

**Figure 3:**
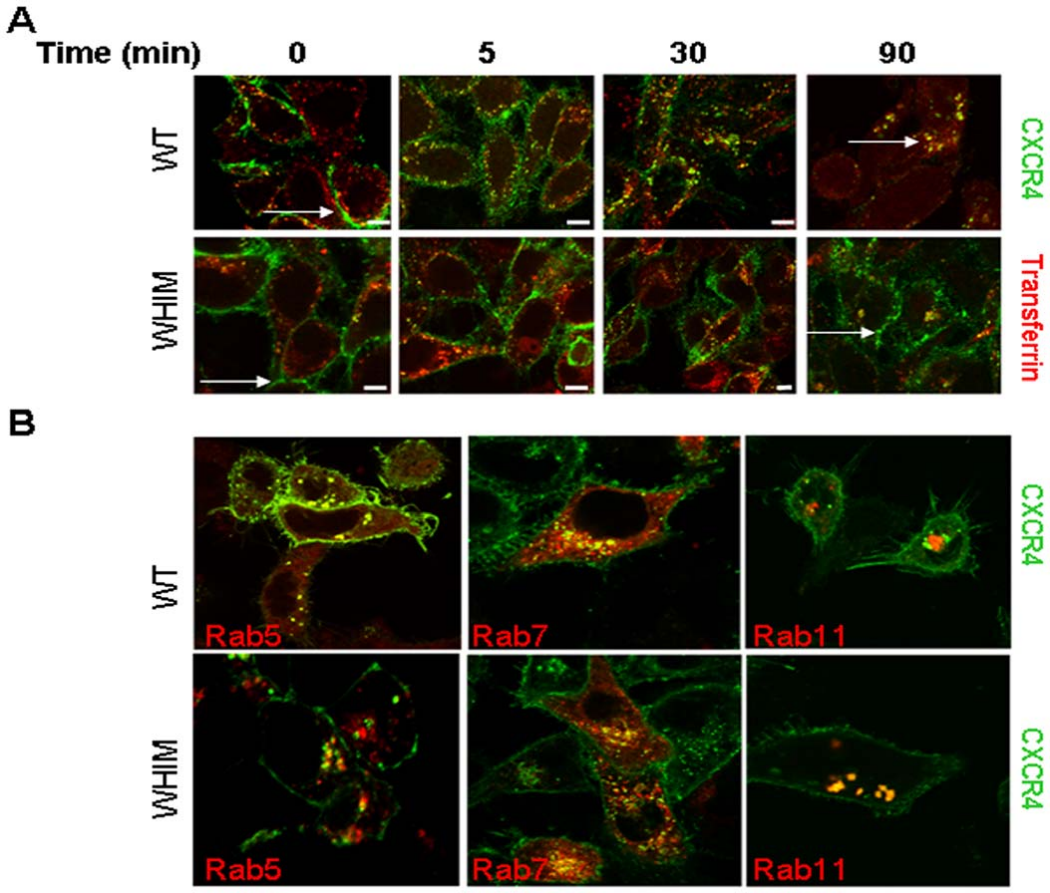
Analysis of trafficking pathways utilized by WT and WHIM mutant CXCR4. **A**, HeLa cells stably expressing GFP-WT CXCR4 or GFP-WHIM CXCR4 were incubated at 37°C with SDF-1/CXCL12 (25 ng/ml) for 0, 5, 30 and 90 min in the presence of labeled (Alexa 568) transferrin and anti-GFP antibodies. The cells were fixed, immunostained with anti-GFP antibodies and examined by confocal microscopy. Representative images depicting the distribution of internalized CXCR4 (green), internalized transferrin (red) and colocalization of internalized CXCR4 and transferrin (yellow). The white arrows highlight the different degree of receptor internalization by the WT and the WHIM CXCR4 at time 0 and 90 min. **B**, HeLa cells stably expressing GFP-WT CXCR4 or GFP-WHIM CXCR4 were transfected with plasmids encoding Rab11-DsRed2, Rab5-RFP, or Rab7-RFP, incubated at 37°C overnight, fixed and mounted.

The defect in ligand-induced internalization exhibited by the WHIM mutant receptor could also be visualized by immunofluorescence using confocal microscopy. We incubated the cells at 4°C with anti-GFP antibodies to distinguish the transfected receptor on the cell surface from the receptor contained within internal organelles. After washing, the cells were incubated with labeled transferrin and either fixed or incubated at 37°C for the times indicated in the presence of the ligand (10 ng/ml) to induce receptor internalization, and then washed and fixed. We observed a clear difference between cells expressing the WHIM mutant and WT receptor in the degree of ligand-induced CXCR4 internalization. After 90 minutes incubation, most of the WHIM mutant was detected on or close to the cell surface membrane (Figure 3A, white arrows), whereas most of the WT receptor was detected inside the cells (Figure 3A, white arrows). At time 0, both WT and mutant receptors were detected on or close to the cell surface membrane (Figure 3A, white arrows).

A possible explanation for the kinetic difference in receptor internalization is that the WT and mutant receptors use a different pathway for internalization. It was previously shown that CXCR4 uses a clathrin-dependent pathway to reach early endosomes [39], [40]. We used immunofluorescence to examine co-localization of the WT and WHIM mutant receptors with the endosomal markers: Rab5, Rab7 and Rab11 [40]. As can be seen in figure 3B, there was no discernible difference in trafficking patterns between the WT and mutant receptor as both receptors colocalized with the three endosomal markers. These results provide evidence for a common trafficking pathway of the WT and WHIM mutant receptors.

### Recruitment of β-Arrestin 2 but Not β-Arrestin 1 Is Delayed to the WHIM CXCR4 Receptor

**Figure 4:**
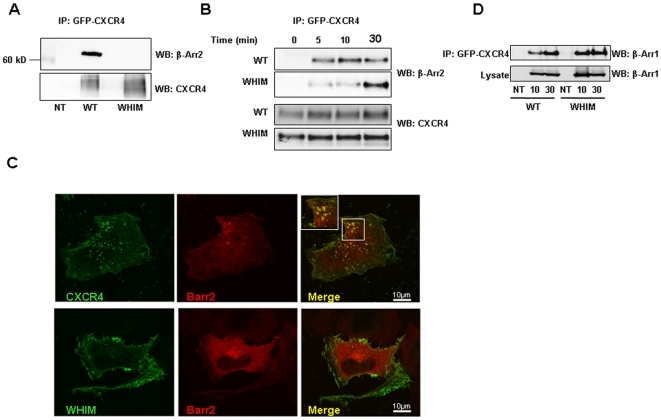
Analysis of WT-CXCR4 and WHIM-CXCR4 interaction with β-Arrestin 2. **A**, Non transduced (NT) Hela cells, or HeLa cells stably expressing GFP-WT CXCR4 or GFP-WHIM CXCR4 were transiently transfected with a FLAG-tagged β-Arrestin 2 construct; 48 hours after transfection, the cells were incubated at 37°C with SDF-1/CXCL12 (25 ng/ml) for 10 min; cell lysates were immunoprecipitated with anti-GFP antibodies to detect transduced CXCR4; the immunoprecipitates were separated through SDS-PAGE and analyzed by Western blotting. The results reflect immunoblotting with anti-FLAG antibodies to detect β-Arrestin 2 and reprobing with antibodies to GFP to detect the immunoprecipitated GFP-CXCR4 (representative from 5 experiments). **B**, The experiment was performed as in (A), except that the HeLa cells were exposed to SDF-1/CXCL12 for 5, 10 and 30 min prior to lysis. **C**, HeLa cells stably expressing GFP-WT CXCR4 or GFP-WHIM CXCR4 were transiently transfected with FLAG-tagged β-Arrestin 2, incubated with 25 ng/ml SDF-1/CXCL12 for 10 min or 30 min at 37°C, fixed and then stained with anti-FLAG antibody to detect β-Arrestin 2 (Barr2, red). GFP fluorescence (green) denotes the transduced CXCR4; colocalization of β-Arrestin 2 and CXCR4 is in yellow. Each of the images shown is representative of 10 fields examined. The experiment is representative of 3 independent experiments performed. **D**, Hela cells stably expressing GFP-WT CXCR4 or GFP-WHIM CXCR4 were either mock transfected (NT) or were transfected with a FLAG-β-Arrestin 1 construct; after 48 hours the cells were exposed to SDF-1/CXCL12 (25 ng/ml, 37°C, 10 and 30 min) and the cell lysates were immunoprecipitated with anti-GFP antibodies. Immunoprecipitates and aliquots of cell lysates used for the IP were separated onto SDS-PAGE and immunoblotted with anti-FLAG antibodies to detect β-Arrestin 1.

Earlier studies have demonstrated that ligand-activated CXCR4 binds β-Arrestin 2 (also known as Arrestin 3), a process that facilitates receptor internalization [27], [41]. Recently, a GFP-tagged β-Arrestin 2 was reported to physically interact with the WT and a WHIM-associated CXCR4 mutant receptor (CXCR4^1013^), providing evidence that a 15-residue C-terminal truncation of CXCR4 does not prevent β-Arrestin 2 binding to the receptor [31]. In addition, since β-Arrestin 2 silencing normalized the extended ligand-induced Erk 1/2 activation by this CXCR4^1013^mutant receptor, β-Arrestin 2 was implicated in this defective response [31]. To further evaluate the potential role of β-Arrestin 2 in the kinetic abnormalities of ligand-induced WHIM mutant CXCR4 activation, we transfected HeLa cells (parental, WT GFP-CXCR4 and WHIM GFP-CXCR4, which lacks the 19 C-terminal residues) with a FLAG-tagged construct of β-Arrestin 2. First, we examined whether the FLAG-β-Arrestin 2 could associate with WT and WHIM CXCR4. After stimulating the HeLa cells for 10 min with CXCL12/SDF-1, immunoprecipitating CXCR4 (anti-GFP antibody) and re-blotting for β-Arrestin 2 (anti-FLAG antibody), we detected β-Arrestin 2 (βarr2) in immunoprecipitates from HeLa cells transduced with the WT receptor, but not from HeLa cells transduced with the WHIM mutant receptor (Figure 4A). This reproducible difference could not be attributed to reduced immunoprecipitation of WHIM mutant CXCR4 compared to the WT receptor from HeLa cells (Figure 4A). The immunoprecitation was specific, as GFP-CXCR4 was not detected in cell lysates from the parental, non-transduced (NT) Hela cells (Figure 4A).

This result was unexpected because Lagane et al. reported that a WHIM mutant receptor with a similar C-terminal truncation (15 residues compared to 19 residues used here) associated with β-Arrestin 2 [31]. Since Lagane et al. reported examining one time point only, which was not described [31], we evaluated whether this difference might be attributable to a kinetic difference. To test for this possibility, we exposed HeLa cells to SDF-1/CXCL12 for different intervals, immunoprecipitated CXCR4 by using an anti-GFP antibody and probed the immunoprecipitates with an antibody to β-Arrestin 2. In the absence of ligand, no FLAG-β-Arrestin 2 could be immunoprecipitated from Hela cells expressing WT or WHIM mutant CXCR4 (Figure 4B). Upon addition of SDF-1/CXCL12 for 5, 10 and 30 minutes, the WT and mutant receptors showed kinetic differences in their ability to associate with β-Arrestin 2, as judged by co-precipitation: at 5 and 10 minutes, the WT CXCR4 was complexed with β-Arrestin 2, but the mutant CXCR4 was minimally associated (Figure 4B). Only after 30 minutes exposure to the ligand, did we detect the mutant CXCR4 in a complex with β-Arrestin 2 (Figure 4B). While confirming the earlier observation that mutant CXCR4 can associate with β-Arrestin 2, the current biochemical data uncovered kinetic differences in the recruitment of β-Arrestin 2 by the WT and WHIM mutant CXCR4. Such kinetic difference is consistent with the results (Figures 1 and 2) showing that the mutant receptor is slower at leaving the plasma membrane in the presence of ligand.

We confirmed these results by immunofluorescence staining and confocal microscopy. CXCR4-expressing (WT or WHIM mutant) HeLa cells were transiently tranfected with a FLAG-tagged β-Arrestin 2 construct, and 3 days later the cells were incubated for 10 minutes with CXCL12/SDF-1. In the case of cells expressing WT-CXCR4, β-Arrestin 2 (Barr2) could to be seen in punctate structures that colocalized at least in part with the receptor (Figure 4C, representative images of 10 fields examined). By contrast, in the case of cells expressing the WHIM mutant receptor, β-Arrestin 2 was not detected in such structures after 10 minutes exposure to the ligand, and the mutant receptor was mostly confined to the cell surface (Figure 4C, representative images of 10 fields examined).

The proteins β-Arrestin-2 (also known as Arrestin-3) and β-Arrestin-1 (also known as Arrestin 2) share a 78% identity at the amino acid level, and have both been shown to play a role in the internalization of various seven-membrane spanning receptors, including CXCR4 [42]. Therefore, we examined whether the delayed recruitment of β-Arrestin 2 by the mutant WHIM CXCR4 receptor was accompanied by a delay in the recruitment of β-Arrestin 1. In co-immunoprecipitation experiments, we found that β-Arrestin-1 similarly associated with the WT and WHIM mutant CXCR4 receptors after 10 and 30 minutes activation by the ligand (Figure 4D). These experiments provide evidence that delayed binding to WHIM-mutant CXCR4 is a characteristic of β-Arrestin 2, but not β-Arrestin 1.

### Grk6 and Grk3 Associate with CXCR4

G protein-coupled receptor kinases (Grks) phosphorylate ligand-activated G protein-coupled receptors on serine and threonine residues within the carboxy-terminal tail and intracellular loops, a process that is accompanied by the recruitment of β-Arrestins [27]. Among the seven known Grks [41], there is circumstantial evidence that Grk6 and Grk3, but not other Grks, may participate in WHIM-associated defective CXCR4 internalization. In Grk6-deficient mice, the neutrophils display enhanced SDF-1/CXCL12-induced chemotaxis in vitro, and thus display some of the hematological abnormalities of patients with WHIM syndrome [33], [43]. In addition, Grk3 was shown to complex with CXCR4 [44]. Recently, a patient with WHIM syndrome who did not have a mutant CXCR4 (WHIM ^WT^) was reported to have a selective decrease in GRK3 expression levels [32]. Thus, Grk3 deficiency has been associated with WHIM syndrome [32]. Despite these important findings, the role of Grk3 and Grk6 in physiological CXCR4 internalization and WHIM-mutant CXCR4 dysfunction is still unclear.

**Figure 5:**
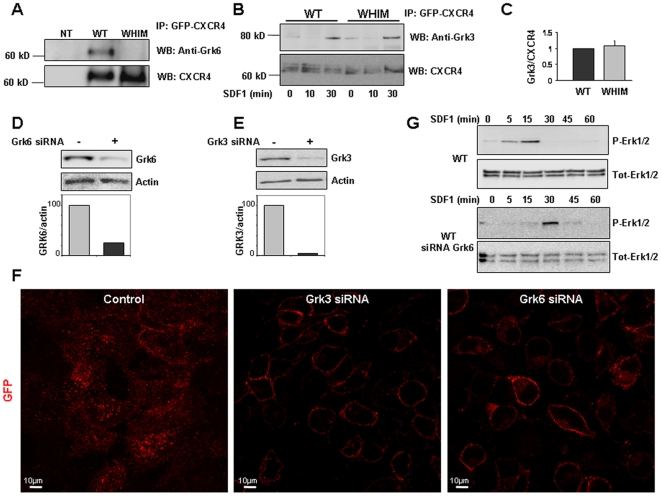
CXCR4 interaction with Grk3 and GRK6. **A**, HeLa cells expressing GFP-CXCR4 (WT or WHIM mutant) were incubated with SDF-1/CXCL12 (25 ng/ml, 37°C, 30 min); cell lysates were immunoprecipitated with anti-GFP antibodies to immunoprecipitate GFP-CXCR4; the immunoprecipitates were separated by SDS-PAGE and immunoblotted with an antibody to Grk6. The blots were re-probed with anti-GFP antibodies to detect transduced CXCR4. **B** and **C**, Hela cells expressing GFP-CXCR4 (WT and WHIM) constructs were incubated with SDF-1/CXCL12 (25 ng/ml, 37°C, 30 min) immunoprecipitated with anti-GFP antibody to pull down transduced CXCR4, probed for Grk3, and reprobed for CXCR4 with anti-CXCR4 antibodies. Representative results are shown in B; average ratio (±SD) of Grk3 and CXCR4 band intensity in 3 separate immunoprecipitation experiments shown in C. **D** and **E**, levels of Grk6 (D) or Grk3 (E) in HeLa cells expressing GFP-WT CXCR4 after 3-day treatment with control or specific siRNAs detected by immunoblotting with antibodies to Grk3 or Grk6. The membranes were reprobed with anti-actin antibodies. **F**, Representative images from confocal microscopy showing internalization of cell surface GFP-CXCR4 after treatment with SDF-1/CXCR4 (25 ng/ml, 37°C, 30 min) in the presence of anti-GFP antibodies. The HeLa cells were either treated with control siRNA or depleted of Grk3 or Grk6 using specific siRNA. **G**, HeLa cells expressing GFP-WT CXCR4 were transfected with scrambled or siRNAs specific to Grk6, and exposed to SDF-1/CXCL12 (25 ng/ml) for the indicated time intervals. Cell lysates were separated by SDS-PAGE and analyzed by Western blotting using specific antibodies to phosphorylated pErk 1/2; membranes were re-probed with antibodies to total Erk 1/2 for loading controls.

To identify whether Grk3 or Grk6 might be involved in the CXCR4 responses to SDF-1/CXCL12, we first examined expression of Grk3 and Grk6. By Western blotting, we detected similar levels of Grk3 and Grk6 in HeLa cells stably expressing GFP-WT or GFP-mutant CXCR4 (Figure S1). We then performed co-immunoprecipitation experiments in the presence or absence of SDF-1/CXCL12 (25 ng/ml, 10 minutes, 37°C). We used antibodies to GFP to immunoprecipitate CXCR4 from HeLa cells stably expressing GFP-WT or GFP-mutant CXCR4, and probed the precipitates with antibodies to Grk6 and Grk3. We found both kinases to be associated with ligand-activated WT CXCR4 (Figure 5A and B). When the WHIM mutant CXCR4 was used in the co-immunoprecipitation assays, we found no receptor association with Grk6 (Figure 5A). By contrast, the ligand-activated WHIM mutant CXCR4 and the WT receptors bound similarly to Grk3 (Figure 5B). This observation was confirmed in 3 separate experiments (Figure 5C). These results suggest that the C-terminal 19 residues of CXCR4 are important for the interaction with Grk6 but not Grk3, and provide evidence that Grk3 and Grk6 may not require the same CXCR4 structural elements for interaction.

### Silencing Grk6 and Grk3 Impairs CXCR4 Trafficking

Depletion of β-Arrestin 1 was reported to cause CXCR4 trapping in early endosomes, which was attributed to defective recruitment of the ubiquitin ligase AIP4 (atrophin1 interacting protein 4) [39]. If Grk3 and Grk6 play a role in recruiting the β-Arrestins to the CXCR4 receptor, as previously suggested [27], then silencing the two kinases should provide a similar phenotype to that derived from silencing β-Arrestin 1. To test for this possibility, we examined the effects of silencing Grk3 or Grk6 in HeLa cells on ligand-induced CXCR4 receptor internalization. Using siRNA oligos, we substantially depleted the cells of Grk3 or Grk6 (Figure 5D and E). By immunofluorescence, we observed (Figure 5F) significant CXCR4 receptor (marked by GFP) internalization in control-treated HeLa cells. By contrast, when HeLa cells were depleted of either Grk3 or Grk6 we observed CXCR4 receptor staying at or close to the plasma membrane. These results provide evidence for a role for both Grk3 and Grk6 in CXCR4 trafficking.

### Grk6 Can Influence CXCR4 Signaling

Lefkowtiz and colleagues [45] have shown that β-Arrestin 2 can serve as a scaffolding protein for signaling molecules while Lagane et al. [31] and others have shown that β-Arrestin 2 is important for Erk 1/2 signaling through CXCR4. If Grk6 is important for β-Arrestin 2 recruitment to CXCR4 then silencing Grk6 should alter receptor signaling. We tested this hypothesis by comparing pErk 1/2 signaling in Grk6-silenced and control Hela cells expressing WT-CXCR4. As shown in Figure 5G, Grk6 depletion led to a delay of pErk 1/2 activation by SDF1, which was similar to that seen in Hela cells expressing the WHIM-CXCR4 receptor (compare with Figure 2D). This is further evidence that Grk6 is important in CXCR4 function.

## Discussion

In the current study, we have dissected the early events that follow ligand binding to the CXCR4 receptor to identify deficiencies stemming from a mutant CXCR4, which is missing the 19 carboxy-terminal residues and is associated with WHIM syndrome. Five important observations were made. First, the mutant receptor is internalized later than the WT receptor after ligand binding. Second, the mutant and WT receptors use the same protein trafficking pathway marked by Rab 5, 7, and 11. Third, ligand-induced phosphorylation of Erk 1/2 is delayed in cells bearing the mutant receptors. Thus, the presence of both a WT and WHIM mutant allele would be expected to result in prolonged Erk 1/2 activation stemming from the combined effect of WT and mutant receptor. Fourth, β-Arrestin 2 recruitment to the activated mutant receptor is delayed compared to the WT receptor, whereas β-Arrestin 1 is not. Fifth, Grk3 and Grk6 bind to the active WT receptor and both are important for its trafficking. However, Grk6 fails to physically associate with the ligand-activated mutant receptor.

It was previously noted that activation of the Erk1/2 signaling pathway is altered in primary cells from a WHIM patient carrying a 15-residue C-terminal CXCR4 truncation and in cells transduced with this mutant receptor [31]. Additionally, it was previously proposed that β-Arrestin 2 contributes to altered signaling by WHIM-mutant CXCR4 receptor [31], [33]. However, these earlier studies emphasized the enhanced/prolonged signaling by the mutant receptor and its prolonged association with β-Arrestin 2 in comparison with the WT receptor. Our experiments disclose the absence of physical interaction between Grk6 to the activated mutant receptor, a substantial delay in recruitment of β-Arrestin 2 to the activated mutant receptor, and a delay in receptor internalization, trafficking and Erk1/2 activation induced by the ligand. Thus, our results explain the abnormally prolonged ligand-induced WHIM receptor signaling and impaired receptor desensitization reported previously [31] on the basis of a combined effect of WT and WHIM CXCR4 molecules rather than a unique dysfunction of the mutant receptor.

Grk6-null mice display markedly reduced neutrophil mobilization from the bone marrow to the peripheral blood in response to G-CSF in spite of having normal hematopoiesis. Thus, Grk6 deficiency in mice reflects some of the characteristically defective neutrophil mobilization of patients with WHIM [19], [20]. In vitro studies with cells from Grk6-null mice showed that splenocytes have increased receptor “sensitivity” to CXCL12/SDF-1 as assessed by GTPase activity [33], and bone marrow-derived neutrophils display increased chemotactic responses to CXCRL12/SDF-1. WHIM leukocytes also have enhanced responses to CXCL12/SDF-1 [30], [31], much like Grk6-null neutrophils from the mice. Thus, previous observations support a role for Grk6 as a regulator of CXCR4 function and a mediator of receptor dysfunction in WHIM. However, one important distinction between WHIM patients and Grk6-null mice is that the mice did not show a difference in blood neutrophil levels compared to WT mice, suggesting that Grk6 alone does not control neutrophil release from the bone marrow. Interestingly, a WHIM patient expressing WT CXCR4 was reported to have reduced expression of Grk3, suggesting that Grk3 deficiency alone may be responsible for CXCR4 dysfunction [32]. Our results suggesting that Grk3 and Grk6 may bind to CXCR4 in different locations are consistent with the possibility that functional defects in each of these kinases may be associated with CXCR4 dysfunction and disease. Previously, Grk3 was reported to bind to CXCR4, but the sites were not further defined [44]. Other studies have disclosed that the cytoplasmic loops or the tail in different G protein-coupled receptors can bind different Grks, but these regions are not conserved between receptors and sequences conferring Grk-binding activity are currently poorly defined [46], [47]. We now found that CXCR4 lacking 19 carboxy-terminal residues is defective at recruiting Grk6, but not Grk3.

Grk6 contributes to CXCR4 phosphorylation [44], and presumably the WHIM-CXCR4 mutant is phosphorylated to a lower degree than the WT receptor, a result reported by Orsini et al. [41]. Indeed, various CXCR4 mutants within the carboxy-terminal tail, including a mutant lacking the terminal 7 residues, resulted in reduced CXCR4 phosphorylation after activation [41]. An important question is whether a direct relationship exists between the failure of Grk6 to associate with the WHIM CXCR4 receptor and the observed delay in β-Arrestin 2 recruitment. Many studies have shown that once phosphorylated, G protein-coupled receptors bind Arrestins through multiple interactions [27]. CXCR4 can bind β-Arrestin 2 through the third intracellular loop and the C-terminal tail [27], [48]. In particular, serine clusters missing from the WHIM mutant receptor may serve to stabilize CXCR4 interactions with β-Arrestins [27], and contribute to β-Arrestin 2 regulation of CXCR4 [48].

Previously, β-Arrestin 1 was shown to contribute to CXCR4 internalization and signaling in response to CXCL12/SDF-1 [48], and to be required for recruitment of the ubiquitin ligase AIP4 and eventual lysosomal trafficking of the CXCR4 receptor [39]. We show that the WT and WHIM receptors can both associate with β-Arrestin 1, and we find no difference in the kinetics of β-Arrestin 1 interaction with WT versus mutant CXCR4 receptors. These results are consistent with the notion that β-Arrestin 1 and β-Arrestin 2 have independent functions [42], [49], but does not exclude the possibility that β-Arrestin 1 may play some indirect role in WHIM-CXCR4 deficiencies. Of note, Cheng et al [48] observed that β-Arrestin 2 was more dependent on CXCR4 phosphorylation then β-Arrestin 1, an observation supported by our data showing that a receptor that lacks an interaction with Grk6 also shows aberrant β-Arrestin 2 recruitment. It is possible that β-Arrestin 2, once it is recruited to the active CXCR4 [31], [48] may indirectly regulate the phosphorylation of β-Arrestin 1 through the downstream mediators phospho-Erk 1/2 [50], and by this mechanism modulate the recruitment of clathrin and/or the ubiquitin ligase AIP4 [39], [51]. Noteworthy, CXCR4 sorting to lysosomes is mediated through ubiquitination of lysine residues included in the “degradation motif” of CXCR4, which spans residues 324–333 [51], a region that is preserved in the WHIM 334-mutant receptor.

Several studies have reported the formation of CXCR4 dimers or heterodimers [31], [52]–[54]. It is unclear how dimerization may influence the receptor's ability to bind ligand and signal. In the case of WHIM patient cells, where both a WT and mutant form exist, it is possible that extended signaling could result from either individual receptor signaling, or alternatively from a single signaling unit comprising heterodimeric WT and mutant CXCR4. Further work will be required to distinguish between these two possibilities. Regardless of whether the functional receptor unit is a dimer or a monomer, our results clearly show the critical role of Grk6-CXCR4 receptor interaction for proper receptor function.

Most of our studies were performed in the well-characterized Hela cells, but many of the results were confirmed in neuroblastoma cells and myeloid cells. Nonetheless, it is possible that subtle differences may exist among different cell types depending on receptor and effectors expression levels. In conclusion, we present biochemical evidence for the cause of WHIM disease associated with mutant CXCR4. In addition, our findings provide further insight into the complex interactions that mediate CXCR4 signaling.

## Materials and Methods

### Cells and Cell Culture

HeLa and HEK 293 cells (American Type Culture Collection, ATCC) were propagated in Dulbecco's Modified Eagles Medium (DMEM, Gibco) supplemented with 10% heat-inactivated Fetal Bovine Serum (FBS, Atlanta Biologicals), glutamine and penicillin/streptomycin (Gibco). Stable cell lines expressing recombinant CXCR4 were produced by transfecting HeLa cells with an expression plasmid for GFP-tagged human wild-type (WT) or mutant CXCR4 using Lipofectamine Reagent (Invitrogen) with 5 µg of DNA/10 cm tissue culture plate (BD Biosciences), according to the manufacturer's instructions. The transfected cells were selected by culture in the presence of G418 (5 µg/ml). The GFP-expressing cells were sorted by flow cytometry (FACS). Transient transfections of WT and WHIM CXCR4 plasmids in HEK 293 and HeLa cells grown in six well tissue culture plates (BD Biosciences) were performed with Lipfectamine 2000 reagent (Invitrogen) according to the manufacturer's protocol. The pre-myeloid cell line KG1a was a kind gift from Jennifer Lippincott-Schwartz (NICHD, NIH Bethesda, MD). Using the retrovirus LZRSpBMN-linker-IRES-eGFP plasmid expressing WT CXCR4 or WHIM CXCR4, KG1a cells were infected with a retrovirus as described [26], and sorted by FACS for GFP expression.

### Reagents

Recombinant human SDF-1/CXCL12 was purchased from R&D Systems or PeProtech, Inc. The following antibodies were used: anti-GFP (Covance Research Products MMS-118P), anti-Grk6 (Abnova 8D4), anti-Grk3 (Abcam ab38294), anti-FLAG (M2, Sigma). All secondary HRP-conjugated antibodies were from GE Healthcare. All Dylight conjugated secondary antibodies were from Pierce. Unless otherwise noted, all other reagents were purchased from Sigma-Aldrich.

### Constructs

Human CXCR4 was cloned into the pPCR-Amp Script vector (Stratagene) by PCR amplification from peripheral blood of a healthy adult individual or a WHIM patient diagnosed with the previously described R334X mutation [22]. After digestion with Xho I and Sac II enzymes, the insert was ligated into the pEGFP-C1 vector (Clontech) at the BamHI and HindIII sites. Accuracy of all constructs was verified by sequencing. The pcDNA3 Barr1-FLAG and pcDNA3 Barr2-FLAG plasmids were a kind gift of Dr. Robert J. Lefkowitz [55] via the Addgene repository (plasmids 14687 and 14685 respectively) [56]. The retrovirus LZRSpBMN-linker-IRES-eGFP expressing either WT or WHIM was constructed by digesting pEGFP-C1-CXCR4-WT or pEGFP-C1-CXCR4-WHIM with XhoI and NotI and ligating the purified fragment into the LZRS vector.

### RNA Interference

The siRNAs for Grk3 and Grk6 (Dharmacon) were transfected individually into cells using Oligofectamine (Invitrogen) following the manufacturer's instructions. The cells were assayed 36 h after transfection.

### Immunoprecipitation

HeLa cells were grown to approximately 75% confluency, and then lysed in 1% (vol/vol) Triton X-100 in 150 mM NaCl, 50 mM Tris-HCl, pH 7.5 at 4°C. The supernatants were precleared for 1 h at 4°C using GammaBind-plus sepharose (GE Healthcare) and were then incubated overnight at 4°C with a primary antibody followed by GammaBind-plus sepharose for 1 h. The beads were spun down and the supernatants removed. The beads were then washed in wash buffer (150 mM NaCL, 0.1% Triton X-100, 50 mM Tris HCl pH 7.5) and run through a 4–20% gradient gel (NuPage), transferred to nitrocellulose and immunoblotted with various antibodies.

### Immunoblotting

Cells were lysed by using 1% (vol/vol) Triton X-100 in 150 mM NaCl, 50 mM Tris-HCl, pH 7.5 at 4°C. The cells were centrifuged at 13,000 rpm for 5 min to remove insoluble material, and NuPage sample buffer was added to the supernatant. The samples were separated by SDS-PAGE (10–20% NuPage). The proteins were then transferred to nitrocellulose membranes. Membranes were blocked for 1 h in TBS with 5% milk (wt/vol) and 0.5% Tween 20 or in TBS with 0.5% BSA (wt/vol) and 0.5% Tween 20 if phospho antibodies were used. Primary antibodies were added in blocking buffer and incubated at 4°C overnight. The membranes were washed four times with TBS-T (TBS plus 0.05% Tween 20). The secondary antibody was incubated for 1 h in blocking buffer. The membranes were then washed again in TBS-T, and proteins were detected by using SuperSignal West (Pierce).

### Uptake and Transferrin Internalization Assay

HeLa cells were grown on glass coverslips (12 mm) to approximately 75% confluency. The cells were washed with cold DMEM, and then incubated with anti-GFP antibody (1 µg/0.1 ml) and/or Transferrin-Alexa 568 (5 µg/0.1 ml, Invitrogen) on ice for 1 h. The coverslips were then washed three times with cold DMEM and then incubated at 37°C with 25 ng/ml SDF1/CXCL12 for the times indicated. The cells were then washed with cold PBS and fixed with 4% paraformaldehyde for immunofluorescence staining.

### Immunofluorescence

Cells were grown in DMEM supplemented with 10% FBS, glutamine and penicillin/streptomycin. For immunofluorescence, Hela cells were subcultured onto round 12 mm diameter coverslips (Daigger) and grown overnight prior to transfection; 24 to 48 h post-transfection, the cells were fixed with 4% paraformaldehyde in PBS for 12 min, washed twice in PBS for 5 min each and then incubated with the appropriate primary antibody in a solution of 0.1% saponin, 0.02% sodium azide and 0.1% Fish Skin Gelatin in PBS for 1 h. The coverslips were then incubated with the appropriate secondary antibody conjugated to either DyLight 549 or DyLight 633 (Pierce) diluted in the same solution used for primary antibodies and incubated for 1 h. The coverslips were then mounted onto glass slides with Fluoromount G (Southern Biotech) and sealed with nail polish to be viewed on a Leica SP2 microscope using a 63X objective.

### FACS Analysis

Cells were first washed by using PBS, and harvested by adding 5 mM EDTA in PBS and incubating for 5 min at 37°C. The cells were then washed twice with PBS containing 3% wt/vol BSA. For GFP analysis the cells were analyzed directly. Where indicated, primary antibody was added, and the cells incubated on ice for 1 h. The cells were washed three times with PBS-BSA, and phycoerythrin-conjugated secondary antibody was added for 1 h. Finally, the cells were washed and analyzed by using a three-color FACSCalibur flow cytometer equipped with CELLQUEST PRO software (Becton Dickinson). The data were analyzed using FlowJo (Tree Star) software.

### Attachment Assay

Huvec cells were plated in a monolayer in 96 well flat bottom plates and prepared as described [38]. 1×10^6^ KG1a cells were labeled for 15 minutes at 37°C with 10 µm CFSE in PBS (Invitrogen). The cells were washed with warm media and placed at 37°C for an additional 30 min. The KG1a cells were then added to the 96 well plate containing the Huvec at 1×10^5^ cells in 50 µl per well. At the end of incubation, non adherent cells were removed and adherent KG1a cells counted by measuring fluorescence emission at 520 nm.

### Statistical Analysis

Statistical analysis of group differences was evaluated by Student's *t* test; P values of <0.05 were considered significant.

## Supporting Information

Figure S1 Expression levels of Grk proteins in HeLa cell lines. HeLa cells stably transduced with GFP-WT CXCR4 or GFP-WHIM were lysed and analyzed by SDS-PAGE and Western blotting using the noted Grk antibodies.
(1.28 MB TIF)
