# Supplementary figures and images for: Impaired Recruitment of Grk6 and β-Arrestin2 Causes Delayed Internalization and Desensitization of a WHIM Syndrome-Associated CXCR4 Mutant Receptor
Source: PLoS One. 2009 Dec 1;4(12):e8102. doi: 10.1371/journal.pone.0008102 (PMC2779657; doi:10.1371/journal.pone.0008102)

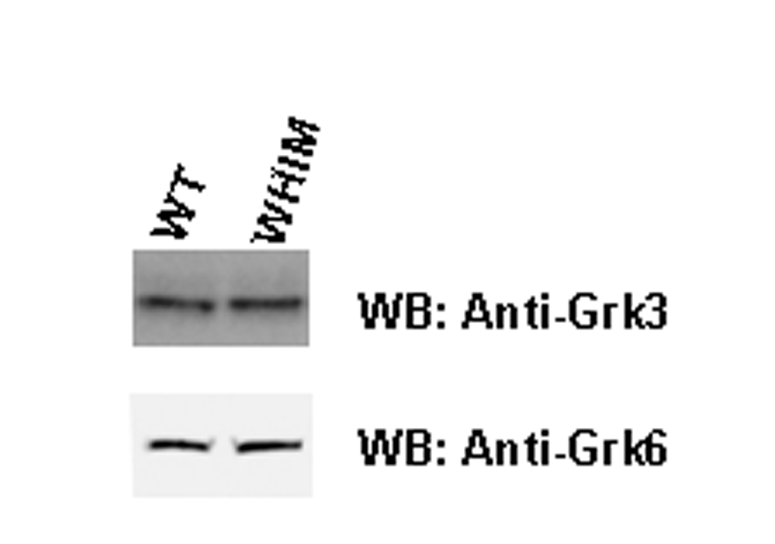

Supplement: Figure S1 — Expression levels of Grk proteins in HeLa cell lines. HeLa cells stably transduced with GFP-WT CXCR4 or GFP-WHIM were lysed and analyzed by SDS-PAGE and Western blotting using the noted Grk antibodies. (1.28 MB TIF) [file pone.0008102.s001.tif]
